# The genome sequence of *Pseudoplusia includens single nucleopolyhedrovirus* and an analysis of *p26* gene evolution in the baculoviruses

**DOI:** 10.1186/s12864-015-1323-9

**Published:** 2015-02-25

**Authors:** Saluana R Craveiro, Peter W Inglis, Roberto C Togawa, Priscila Grynberg, Fernando L Melo, Zilda Maria A Ribeiro, Bergmann M Ribeiro, Sônia N Báo, Maria Elita B Castro

**Affiliations:** Embrapa Recursos Genéticos e Biotecnologia, Parque Estação Biológica, W5 Norte Final, 70770-917, Brasília, DF Brazil; Departamento de Biologia Celular, Universidade de Brasília-UnB, Brasília, DF Brazil

## Abstract

**Background:**

*Pseudoplusia includens single nucleopolyhedrovirus* (PsinSNPV-IE) is a baculovirus recently identified in our laboratory, with high pathogenicity to the soybean looper, *Chrysodeixis includens* (Lepidoptera: *Noctuidae*) (Walker, 1858). In Brazil, the *C. includens* caterpillar is an emerging pest and has caused significant losses in soybean and cotton crops. The PsinSNPV genome was determined and the phylogeny of the *p26* gene within the family *Baculoviridae* was investigated.

**Results:**

The complete genome of PsinSNPV was sequenced (Roche 454 GS FLX – Titanium platform), annotated and compared with other Alphabaculoviruses, displaying a genome apparently different from other baculoviruses so far sequenced. The circular double-stranded DNA genome is 139,132 bp in length, with a GC content of 39.3 % and contains 141 open reading frames (ORFs). PsinSNPV possesses the 37 conserved baculovirus core genes, 102 genes found in other baculoviruses and 2 unique ORFs. Two *baculovirus repeat ORFs* (*bro*) homologs, *bro-a* (Psin33) and *bro-b* (Psin69), were identified and compared with *Chrysodeixis chalcites nucleopolyhedrovirus* (ChchNPV) and *Trichoplusia ni single nucleopolyhedrovirus* (TnSNPV) *bro* genes and showed high similarity, suggesting that these genes may be derived from an ancestor common to these viruses. The homologous repeats (*hrs*) are absent from the PsinSNPV genome, which is also the case in ChchNPV and TnSNPV. Two *p26* gene homologs (*p26a* and *p26b*) were found in the PsinSNPV genome. P26 is thought to be required for optimal virion occlusion in the occlusion bodies (OBs), but its function is not well characterized. The P26 phylogenetic tree suggests that this gene was obtained from three independent acquisition events within the *Baculoviridae* family. The presence of a signal peptide only in the PsinSNPV *p26a*/ORF-20 homolog indicates distinct function between the two P26 proteins.

**Conclusions:**

PsinSNPV has a genomic sequence apparently different from other baculoviruses sequenced so far. The complete genome sequence of PsinSNPV will provide a valuable resource, contributing to studies on its molecular biology and functional genomics, and will promote the development of this virus as an effective bioinsecticide.

**Electronic supplementary material:**

The online version of this article (doi:10.1186/s12864-015-1323-9) contains supplementary material, which is available to authorized users.

## Background

Baculoviruses are specific pathogens of the insect orders Lepidoptera, Diptera and Hymenoptera and exhibit rod-shaped nucleocapsids embedded in a crystalline protein matrix (occlusion bodies – OBs) composed of polyhedrin in nucleopolyhedroviruses (NPVs) and granulin in granuloviruses (GVs) [1-3]. The replication cycle of the baculoviruses is characterized by production of two viral phenotypes: occlusion derived viruses (ODVs) and budded viruses (BVs). These particles are genotypically identical, but they are morphologically and functionally distinct, with the BVs involved in systemic infection within host larvae (produced in an early phase of infection) and the ODVs involved in the horizontal transmission of the virus in the host population (produced in the late phase of infection) [4]. The *Baculoviridae* family consists of four genera: *Alphabaculovirus* (lepidopteran-specific NPV), *Betabaculovirus* (lepidopteran-specific GV), *Gammabaculovirus* (hymenopteran-specific NPV) and *Deltabaculovirus* (dipteran-specific NPV) [2,5,6]. Alphabaculoviruses can be further divided into Groups I and II based on DNA sequence data and differences in BVs, where the envelope fusogenic protein in Group I is GP64 and in Group II is the fusion (F) protein [7-10].

So far, 64 complete baculovirus genomes are present in GenBank, including many of the Alphabaculoviruses (45) followed by 15 Betabaculoviruses, 3 Gammabaculoviruses and the *Culex nigripalpus* Deltabaculovirus (CuniNPV) (http://www.ncbi.nlm.nih.gov/genomes/GenomesGroup.cgi?taxid=10442). Baculovirus genomes range in size from 81.7 (*Neodiprion lecontei nucleopolyhedrovirus*, NeleNPV) to 178.7 kbp (*Xestia c-nigum granulovirus*, XcGV) with GC content below 50% and containing from 89 (NeleNPV) to 183 (*Pseudaletia unipuncta granulovirus*, PsunGV) predicted ORFs [11]. The gene diversity in baculoviruses has been estimated to be about 900 genes, among which 37 (core genes) may play essential biological functions in the replication cycle [12]. The common genomic features of the *Baculovirida*e family include large double-stranded circular DNA, bidirectionally oriented open reading frames (ORFs) which are distributed on both DNA strands, 37 genes common to all baculoviruses (core genes), promoters that regulate the temporal cascade of gene expression and viral genome replication in the host cell nucleus [13].

The soybean looper, *Chrysodeixis includens* (syn., *Pseudoplusia includens)* (Walker, 1858) (Lepidoptera: Noctuidae, Plusiinae) is a lepidopteran pest with restricted distribution in the Western Hemisphere, occurring from the northern United States to southern South America [14,15]. Soybean, cotton, beans, potatoes, tomatoes, tobacco, sunflower, lettuce, cauliflower, cabbage and okra are the most common crops attacked by *C. includens* [16-21]. However, the polyphagous *C. includens* was found feeding on 73 plant species from 29 different families in Brazil [22]. Until 2003, *Anticarsia gemmatalis* was considered one of the most important pests on soybean and the baculovirus *Anticarsia gemmatalis* MNPV was widely used as a bioinsecticide on approximately two million hectares of soybeans [23]. Recently, *C. includens* has begun to have an economic impact due to its population growth, causing significant losses in soybean production. Among other factors, this was attributed to a decline in natural enemies, which previously controlled the pest, and to development of resistance due to indiscriminate use of chemical pesticides in soybean fields [23]. Other forms of control are therefore required, and for this, new baculoviruses may be strong candidates for the biocontrol of this emerging pest.

*Pseudoplusia includens single nucleopolyhedrovirus* (PsinSNPV) is a Group II *Alphabaculovirus* pathogenic to *C. includens* [24]. Seven PsinSNPV (IA to IG) isolates collected on cotton and soybean crops from Guatemala and Brazil were reported to cause fatal infections in *C. includens* larvae [25]. Evidence of significant genetic variations and different degrees of pathogenicity were observed among the isolates analyzed in our previous studies [24,25]. Other PsinNPV isolates, PsinNPV-USA and PsinNPV-GT, have been reported, but little is known about them [26].

The isolate PsinSNPV-IE was obtained from *C. includens* larvae collected on Brazilian soybean crops and was found to be one of the most virulent against *C. includens* among seven isolates analyzed [25]. In this manuscript, we report the complete sequence and organization of the PsinSNPV-IE genome and speculate on the origin of the *p26* gene within the *Baculoviridae* family by potentially distinct acquisition events. The analysis of the PsinSNPV genome will provide important information for a better understanding of its virulence, evolution and molecular biology. These findings may also contribute to the development of a PsinSNPV bioinsecticide for the control of *C. includens*.

## Results and discussion

### Nucleotide sequence and gene content of the PsinSNPV genome

**Figure 1:**
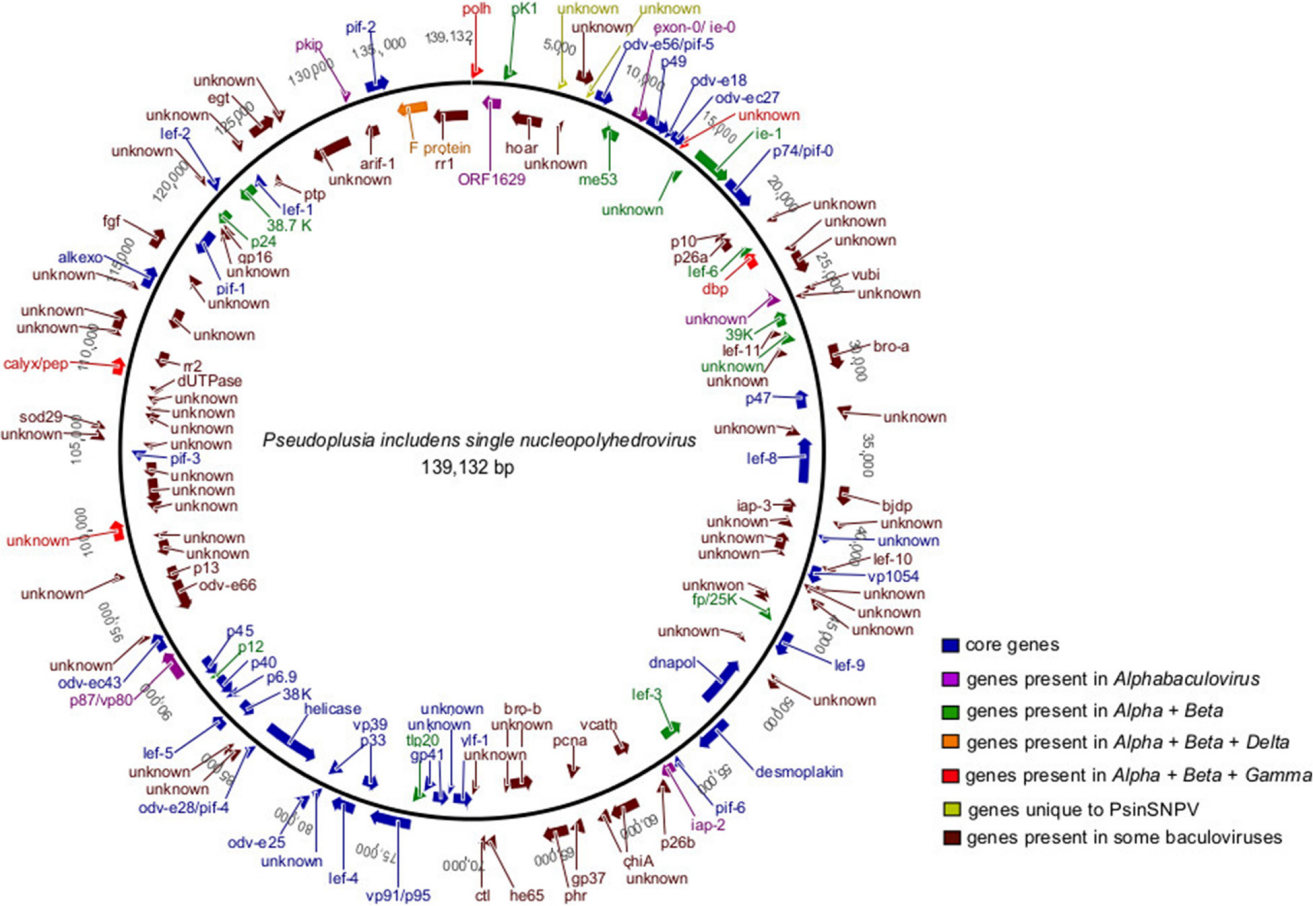
**Circular map of the PsinSNPV genome.** The 141 predicted ORFs and transcription direction are represented by arrows with displayed name. Genome position is indicated in bp, starting from the *polh* gene, by convention.

The PsinSNPV genome was sequenced using next generation technology (NGS) on the Roche 454 GS-FLX Titanium platform. A total of 38,281 reads were obtained with an average length of 542.10 ± 67.48 bp. Pre-processing yielded 33,596 sequences, with a mean length of 350.86 ± 121.32 bp (Additional file 1). Following assembly, the size of the double-stranded circular DNA PsinSNPV genome [GenBank accession number: KJ631622] was determined to be 139,132 bp (30X coverage) with a GC content of 39.3 %, which is in agreement with the average GC content of Group II Alphabaculoviruses (GC = 41.6%) [12]. *In silico* restriction digest analysis of the PsinSNPV genome was conducted, corroborating previous physical restriction maps [25] (data not shown). The ORFs were sequentially numbered starting from the polyhedrin gene in a clockwise orientation. A total of 141 putative ORFs, including the 37 core genes present in all baculoviruses and two PsinSNPV unique ORFs (Psin5 and Psin8), were identified, comprising 80 % of the PsinSNPV genome with 69 ORFs in clockwise, and 72 ORFs in counterclockwise orientation (Figure 1).

### Comparison of PsinSNPV with others Alphabaculoviruses

**Figure 2:**
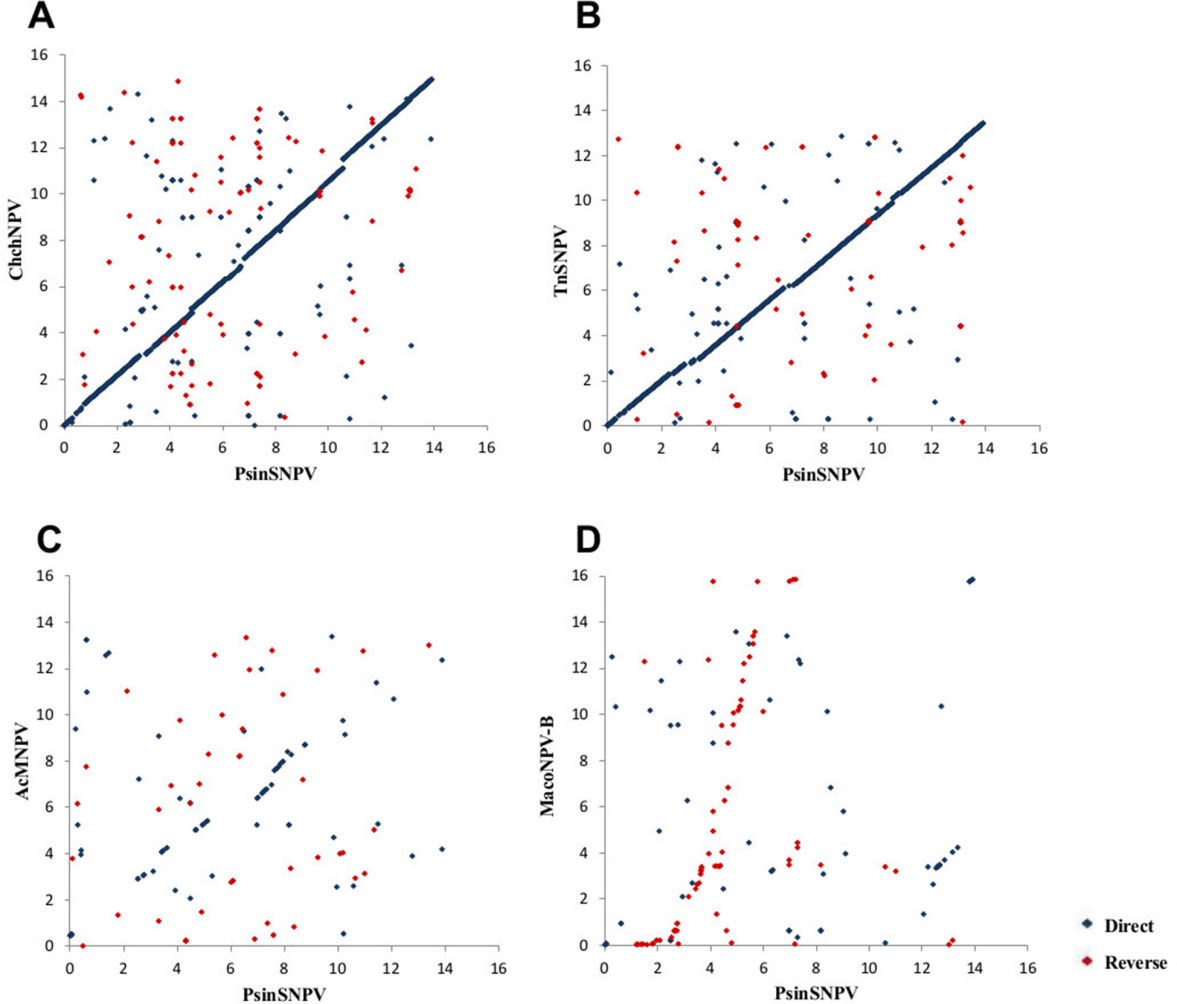
**Dot plot matrix analysis using LBDotView v. 1.0 software.** Blue dots represent the homolog regions of the PsinSNPV genome compared with **(A)** ChchNPV, **(B)** TnSNPV, **(C)** AcMNPV and **(D)** MacoNPV-B genome, both in the direct orientation, and red dots compare the PsinSNPVgenome in the direct orientation with NPV genomes in the reverse orientation.

The PsinSNPV genome was compared with the Alphabaculoviruses ChchNPV, TnSNPV, MacoNPV-B (Group II) and the reference baculovirus, AcMNPV (Group I). The information is summarized in Table 1 and Additional file 2. As expected, the two viruses most closely related to PsinSNPV are ChchNPV and TnSNPV, sharing similar genome sizes and possessing high nucleotide sequence similarity [24]. However, the PsinSNPV genome is 4,738 bp larger than TnSNPV and 10,490 bp smaller than ChchNPV (Table 1). Global alignment and dot matrix analysis of the PsinSNPV genome compared with ChchNPV, TnSNPV, MacoNPV-B and AcMNPV revealed that PsinSNPV is highly similar and collinear with ChchNPV and TnSNPV (70% overall amino acid identity), but not with MacoNPV-B and AcMNPV (50% overall amino acid identity) (Figure 2, Additional file 3 and Table 1). PsinSNPV shares 82 ORFs with AcMNPV, 110 ORFs with MacoMNPV-B, 134 ORFs with ChchNPV and 122 ORFs with TnSNPV (Additional file 2). The ORFs reported as unique in ChchNPV (Chch-24, −34, −36 and −90) and in TnSNPV (Tn-36 and −62) showed similarity to PsinSNPV ORFs-25, −35, −36, −88 and PsinSNPV ORF-40, −65, respectively (Additional file 2).

**Table 1:** **Characteristics of the PsinSNPV genome compared with other Alphabaculoviruses**

**Features**	**Baculoviruses**				
	**PsinSNPV**	**AcMNPV**	**MacoNPV-B**	**ChchNPV**	**TnSNPV**
**Genome size (bp)**	139,132	133,894	158,482	149,622	134,394
**GC content (%)**	39.3	40.7	40.0	39.1	39.0
**ORFs (total)**	140	156	168	151	145
**Coding sequence (%)**	80	91	89	83	95
***hr*** **sequences**	-	9	4	-	-
***bro*** **genes**	2	1	7	4	2
**Mean % aa ID with PsinSNPV**	-	48.6	47.8	73.1	74.5
**Homologs in PsinSNPV**	-	82	110	134	122
**ORFs unique to PsinSNPV**	2	-	-	-	-
**GenBank accession number**	KJ631622	L22858	AY126275	AY864330	DQ017380

### Replication, transcription and structural genes

The baculovirus genes are categorized based on their functions during the viral cycle as follows: DNA replication, RNA transcription, ODV and BV structural proteins or oral infectivity proteins [12]. Baculovirus genome replication mechanisms are still not fully understood. Several studies have been developed to try to identify the genes responsible for DNA replication and translation. The essential DNA replication factors *late expression factor 1* (*lef-1*), *lef-2*, *lef-3*, *DNA polymerase* (*dnapol*), *p6.9*, *38 k*, *helicase* (*hel*) and *immediate early 1* (*ie-1*) homologs are all present in the PsinSNPV genome. In addition, the PsinSNPV genome contains genes homologous to *proliferating cell nuclear antigen* (*pcna*), *major early-transcribed protein 53* (*me53*), *DNA binding protein* (*dbp*), *alkaline exonuclease* (*alkexo*) and *exon-0/ie-0*, which were not identified in all baculoviruses but may influence viral DNA replication [11,27].

The AcMNPV transcription system is activated in two main stages. At first, *lef-4*, *lef-8*, *lef-9* and *p47* are transcribed to encode the 4 subunits of the viral RNA polymerase complex [28]. This complex acts on gene transcription and mRNA processing, including capping and polyadenylation. Then, the transcription enhancers *lef-5* and *very late factor 1* (*vlf-1*) are transcribed [29]. All these genes were also found in the PsinSNPV genome. In addition, some supposedly non-essential genes involved in AcMNPV transcription regulation are also present in the PsinSNPV genome: *lef-6*, *lef-11*, *39 K*, *lef-10* and *protein kinase 1* (*pk1*)*.*

The PsinSNPV genome has 28 known baculovirus genes coding for structural proteins. Genes for ODV and BV structural proteins include *polyhedrin* (*polh*), *orf1629*, *pk1*, *occlusion derived virus envelope protein 18* (*odv-e18*), *occlusion derived virus enveloped capsid protein 27* (*odv-ec27*), *p10*, *viral protein 1054* (*vp1054*), *few polyhedra protein/25 k* (*fp25k*), *desmoplakin*, *41-kDa glycoprotein* (*gp41*), *telokinin-like peptide 20* (*tlp20*), *viral protein 91* (*vp91/p95*), *vp39*, *p33*, *odv-e25*, *p87/vp80*, *odv-ec43*, *odv-e66*, *p13*, *calyx/polyhedrin enveloped protein* (*calyx/pep*), *p24*, *per os infectivity factor 0* (*p74/pif-0*), *pif-1*, *pif-2*, *pif-3*, *odv-e28/pif-4*, *odv-e56/pif-5* and *fusion* (*f*) *protein*. The six PIF genes which are components of ODVs and are involved in oral infectivity exhibited high sequence similarity to ChchNPV and TnSNPV PIFs. The genus *Alphabaculovirus* is divided into Groups I and II based on gene content, and in particular, the BVs fusion protein: GP64 and F protein, respectively. PsinSNPV, a Group II *Alphabaculovirus*, possesses the expected F protein homolog.

### Nucleotide metabolism and DNA repair

Several Group II Alphabaculoviruses and Betabaculoviruses encode genes involved in nucleotide biosynthesis. PsinSNPV possesses the ribonucleotide reductase (RR) large (RR1) and small (RR2) subunits and the dUTPase protein. These RR proteins are enzymes involved in the formation of deoxyribonucleotides from ribonucleotides [3]. The dUTPase protein is responsible for preventing incorporation of mutagenic dUTP into DNA [3,30]. Poly (ADP-ribose) polymerase (PARP) and poly ADP-ribose glycohydrolase (PARG) are enzymes involved in synthesis of ADP riboses that activate and recruit DNA repair enzymes [31-33]. Although it has been reported that all Group II genomes encode PARG homologs [3], the PsinSNPV genome is notable for its absence.

The CPD photolyase, encoded by the *DNA photolyase* (*phr*) gene, acts at cyclobutane pyrimidine dimers to repair ultraviolet (UV) -induced DNA damage. The *phr* gene was identified in ChchNPV [34,35], TnSNPV [36], *Plusia acuta* NPV (PlacNPV) [26] and *Thysanoplusia orichalcea* NPV-B9 (ThorNPV-B9) [26]. Studies suggest that the *phr* gene is conserved in Group II Alphabaculoviruses that infect lepidopteran insects in the Plusiinae subfamily of the Noctuidae family [26]. However, the *phr* gene was also identified in baculoviruses that infect insects of other subfamilies, such as *Spodoptera litura* GV (SpliGV) (subfamily Hadeninae) [37], *Clanis bilineata* NPV (ClbiNPV) (Sphingidae family) [38], *Apocheima cinerarium* NPV (ApciNPV) (Geometridae family) [39] and *Ampelophaga rubiginosa* NPV (AmpeNPV) (Sphingidae family) (unpublished, 2008) [39].

PsinSNPV belongs to a group where the *phr* gene is conserved and, as expected, its genome encodes a CPD photolyase protein (Psin68). The complete nucleotide sequence of the PsinSNPV *phr* gene is of 1,512 bp with GC % = 36.2%. The deduced PHR amino acid sequence of PsinSNPV, ChchNPV- PHR1, −PHR2, and TnSNPV were aligned, revealing that the PsinSNPV photolyase possesses high identity to TnSNPV and ChchNPV- PHR1 (Additional file 4). Previous studies showed that ChchNPV - PHR1 is not active when tested in an *Escherichia coli* photolyase deficient strain [40]. The active copy (PHR-2) is distinct in its possession of two conserved tryptophan residues, which may be involved in an electron transfer mechanism [40]. In the PsinSNPV photolyase protein, the tryptophan residues are replaced by histidine and tyrosine in positions 368 and 370 aa, respectively (Additional file 4). Therefore, both tryptophans are absent in the PsinSNPV photolyase, suggesting that this protein might not be active. The partial PHR amino acid sequences of PsinNPV –GT1 (EU401912); −GT2 [GenBank: EU682272], PsinNPV - USA [GenBank: EU401913] and PsinSNPV-IA to -IG isolates described in the literature [24,39] were aligned, where PsinNPV-GT1 and PsinNPV – USA isolates showed high similarity to the PsinSNPV-IA to -IG isolates. Interestingly, in contrast to other PsinNPV isolates reported so far, PsinNPV - GT2 possesses a tryptophan residue at position 368, which is thought to be essential for enzyme activity (data not shown). Further studies are needed to confirm and investigate the activity of the PsinSNPV - GT2 photolyase.

### Auxiliary genes

The auxiliary genes *viral ubiquitin* (*vubi*), *viral cathepsin* (*v-cath*), *chitinase* (*chiA*), *37-kDa glycoprotein* (*gp37*), *conotoxin* (*ctl*), *superoxide dismutase 29* (*sod29*), *fibroblast growth factors* (*fgf*), *phosphotyrosine phosphatase* (*ptp*), *ecdysone glucose transferase* (*egt*), *actin rearrangement infectivity factor 1* (*arif-1*), *inhibitor of apoptosis 2* (*iap-2*), *iap-3* and *p35/p49* were found in the PsinSNPV genome. The auxiliary genes are non-essential for DNA replication, translation or viral particle formation. However, these genes confer selective advantages to viruses as has been observed in homologs of PsinSNPV auxiliary genes described in the literature. The activity of the *v-cath* and *chiA* genes is notable in *P. includens* larvae infected with PsinSNPV, where the encoded proteins cause degradation and liquefaction of the host cadaver [41,42]. The *fgf*, *ptp* and *egt* genes were reported to be involved in host hyperactive behaviors, increasing larval motility and preventing the molt to extend insect life, respectively [43]. The *ptp* gene was previously reported to be only present in Group I NPVs [3,43], however this gene is present in the PsinSNPV genome.

### Homologous regions (*hrs*) are absent from the PsinSNPV genome

Homologous regions (*hrs*) are repeated sequences with an imperfect palindromic core that are distributed in the genome as singletons or arranged in tandem. These repeat sequences are present in baculovirus genomes and other closely related invertebrate viruses [29]. These regions act as enhancers of early gene transcription in NPVs and may serve as origins of replication in NPVs and GVs [11]. *Homologous regions* are a common feature found in genomes of the four genera of the *Baculoviridae* family. However, no typical baculoviral *hrs* were found in the PsinSNPV genome, which is also the case in *Buzura supressaria* NPV (BusuNPV) [44], ChchNPV, TnSNPV and *Agrotis segetum* GV (AgseGV) [35,36,44,45].

### PsinSNPV *bro* genes

The PsinSNPV genome sequence contains two *baculovirus repeated* ORFs (*bro* genes), named according to their order in the genome: *bro-a* (ORF-33) and *bro-b* (ORF-69). The *bro* genes commonly occur in Alpha-, Beta- and Gammabaculoviruses, varying in number of copies and length among the viruses [46-50]. These genes were first reported from baculoviruses, but *bro* gene homologs were subsequently identified in other insect dsDNA viruses, such as entomopoxvirus and entomoiridovirus [51-53]. BRO proteins exhibit a highly conserved N-terminal DNA binding domain (BRO-N) in the first 100–150 aa and a variable C-terminal domain (BRO-C) [48,54]. The functions of BRO proteins are not clear, but were proposed to be involved in host DNA replication and/or transcriptional regulation and as a viral replication enhancer in the late phase [46,48,49,54]. Although a deletion of 425 bp (386–811) (~140 aa) is present in the PsinSNPV *bro-b* gene compared with the ChchNPV *bro-b* gene, the genes share 72% identity. The PsinSNPV *bro-a* gene showed higher similarity to the *Lymantria xylina* MNPV *bro-m* gene and the *Mamestra brassicae* MNPV *bro-a* gene with 58 and 53% identity, respectively. In contrast to the PsinSNPV BRO-B protein with one BRO-N domain, PsinSNPV BRO-A protein contains two BRO-N domains [Pfam: PF02498] at amino acid position 14 to 118 and 139 to 234. In addition, the PsinSNPV BRO-A protein contains a domain of unknown function DUF3627 [Pfam: PF12299] in amino acid position 334 to 423. Although PsinSNPV, ChchNPV and TnSNPV are closely related, their *bro* genes do not show high similarity.

### ORFs unique to PsinSNPV

Two putative ORFs, Psin5 and Psin8, were found to be unique to the PsinSNPV genome. These ORFs do not show significant similarity to other previously described baculovirus ORFs and exhibit signature sequences that describe domains predicted by InterProScan 5 [55]. Psin5 is predicted to encode a 172 amino acid (aa) protein with molecular weight of 18.73 kDa and shows low homology to a signal transducer and activator of transcription protein in the avian species, *Pseudopodoces humilis* [GenBank: XP_005533966] (%ID = 80%, cover = 40% and e-value = 3E-12). Psin8 is predicted to encode a 150 aa, 11.9 kDa protein, but shows no significant similarity to any genes in GenBank databases (P > 0.01). The TAAG late promoter motif, combined with a TATA early promoter (TATAAGG motif), was identified about 100 bp upstream of both the Psin5 and Psin8 start codons (5,031 and 7,219 nt, respectively). These promoters are thought to be transcribed both by the host RNA polymerase II and viral RNA polymerases [56], suggesting that these genes could be expressed both early and late in infection. A search was made for protein families, domains and functional sites found in transmembrane domains in the predicted Psin5 and Psin8 proteins. Using TMHMM Server v 2.0, transmembrane helices from amino acid position 79 to 101 and 148 to 170 in Psin5 and 69 to 91 in Psin8 hypothetical proteins were predicted (Additional file 5).

### Two *p26* homologs in PsinSNPV

Two *p26* (Ac136) gene homologs were identified in the PsinSNPV genome. The function of the *p26* gene is not well understood, but studies have shown that deletion of the AcMNPV *p26* gene produced no differences in phenotype from wild-type AcMNPV in cells and in larvae [3,57,58]. However, a combined deletion of *p26*, *p10* and *p74* genes in AcMNPV resulted in polyhedra containing few or no virions [58]. For this reason, *p26* is thought to be required for optimal virion occlusion in the OBs.

One or more copies of the *p26* gene are present in all Group I and II Alphabaculoviruses, except *Spodoptera littoralis multiple nucleopolyhedrovirus*, SpliNPV [GenBank: AF325155]. The viruses with more than one *p26* copy belong to Group II Alphabaculoviruses (Table 2). However, in Group I Alphabaculovirus genome sequences available at GenBank, *Choristoneura fumiferana* (Cf) MNPV, *Choristoneura occidentalis* (Choc) NPV and *Choristoneura rosaceana* (Chro) NPV contain two *p26* homologs (Table 2).

**Table 2:** **Amino acid sequences used in phylogenetic analysis of the P26 amino acid sequence**

			**Position (ORF/Signal peptide)**		
**Alphabaculoviruses**	**Abbreviation**	**GenBank**	**1** ^**a**^	**2** ^**b**^	**3** ^**c**^
**Group I**					
*Autographa californica* NPV	AcMNPV	L22858	136 / No	-	-
*Bombyx mandarina* NPV	BomaNPV	FJ882854	120 / No	-	-
*Bombyx mori* NPV	BmNPV	L33180	121 / No	-	-
*Rachiplusia ou* MNPV	RoMNPV	AY145471	129 / No	-	-
*Antheraea pernyi* NPV	AnpeNPV	DQ486030	127 / No	-	-
*Anticarsia gemmatalis* MNPV	AgMNPV	DQ813662	132 / No	-	-
*Choristoneura fumiferana* DEF MNPV	CfDEFMNPV	AY327402	130 / No	-	-
*Choristoneura fumiferana* MNPV	CfMNPV	AF512031	128 / No	-	7 / Yes
*Choristoneura murinana* NPV	ChmuNPV	KF894742	22 / No	-	-
*Choristoneura occidentalis* NPV	ChocNPV	KC961303	21 / No	-	143 / Yes
*Choristoneura rosaceana* NPV	ChroNPV	KC961304	22 / No	-	145 / Yes
*Epiphyas postvittana* NPV	EppoNPV	AY043265	119 / No	-	-
*Hyphantria cunea* NPV	HycuNPV	AP009046	21 / No	-	-
*Maruca vitrata* NPV	MaviNPV	EF125867	104 / No	-	-
*Orgyia pseudotsugata* MNPV	OpMNPV	U75930	132 / No	-	-
*Plutella xylostella* MNPV	PlxyMNPV	DQ457003	132 / No	-	-
*Thysanoplusia orichalcea* NPV	ThorNPV	JX467702	133 / No	-	-
**Group II**					
*Adoxophyes honmai* NPV	AdhoNPV	AP006270	31 / Yes	-	-
*Adoxophyes orana* NPV	AdorNPV	EU591746	30 / Yes	-	-
*Agrotis ipsilon* MNPV	AgipMNPV	EU839994	149 / Yes	100 / No	-
*Agrotis segetum* NPV	AgseNPV	DQ123841	142 / Yes	94 / No	-
*Apocheima cinerarium* NPV	ApciNPV	FJ914221	102 / Yes	40 / No	-
*Buzura suppressaria* NPV	BusuNPV	KF611977	8 / Yes	52 / No	-
*Chrysodeixis chalcites* NPV	ChchNPV	AY864330	19 / No	63 / No	-
*Clanis bilineata* NPV	ClbiNPV	DQ504428	19 / Yes	61 / No	-
*Ectropis obliqua* NPV	EcobNPV	DQ837165	18 / No	52 / No	-
*Euproctis pseudoconspersa* NPV	EupsNPV	FJ227128	51 / Yes	61 / No	-
*Helicoverpa armigera* MNPV	HearMNPV	EU730893	151 / Yes	101 / No	-
*Helicoverpa armigera* NPV	HearNPV	AF303045	22 / Yes	-	-
*Helicoverpa armigera* NPVG4	HearNPV-G4	AF271059	22 / Yes	-	-
*Helicoverpa armigera* NPV NNg1	HearNPV-NNg1	AP010907	21 / Yes	-	-
*Helicoverpa zea* SNPV	HzSNPV	AF334030	21 / Yes	-	-
*Hemileuca sp*. NPV	HespNPV	KF158713	22 / Yes	60 / No	-
*Leucania separata* NPV	LeseNPV	AY394490	20 / No	-	-
*Lymantria dispar* MNPV	LdMNPV	AF081810	40 / Yes	-	-
*Lymantria xylina* MNPV	LyxyMNPV	GQ202541	36 / Yes	-	-
*Mamestra brassicae* MNPV	MabrMNPV	JQ798165	147 / Yes	99 / No	-
*Mamestra configurata* NPV A	MacoNPV-A	U59461	158 / Yes	109 / No	-
*Mamestra configurata* NPV B	MacoNPV-B	AY126275	157 / Yes	108 / No	
*Orgyia leucostigma* NPV	OrleNPV	EU309041	20 / No	62 / No	
*Spodoptera exigua* MNPV	SeMNPV	AF169823	129 / Yes	87 / No	
*Spodoptera frugiperda* MNPV	SfMNPV	EF035042	131 / Yes	86 / No	
*Spodoptera litura* NPV II	SpliNPV-II	EU780426	135 / Yes	91 / No	
*Trichoplusia ni* SNPV	TnSNPV	DQ017380	19 / Yes	59 / No	

^a^P26 ORF number adjacent to *p10* gene.

^b^P26 ORF number adjacent to *iap-2* gene.

^c^P26 ORF number adjacent to *ptp −1* and −*2* genes.

The *p26* gene is conserved in position, adjacent to the *p10* gene in all the Alphabaculoviruses containing a single copy. The PsinSNPV *p26a*/ORF-20 and *p26b*/ORF-62 are positioned adjacent to the *p10* gene and adjacent to the *iap-2* gene, respectively. The copy adjacent to the *iap-2* gene is also positionally conserved in all Group II Alphabaculoviruses containing two *p26* copies. However, the second *p26* copy in Group I Alphabaculoviruses is positioned adjacent to the *ptp1* and *ptp2* genes.

**Figure 3:**
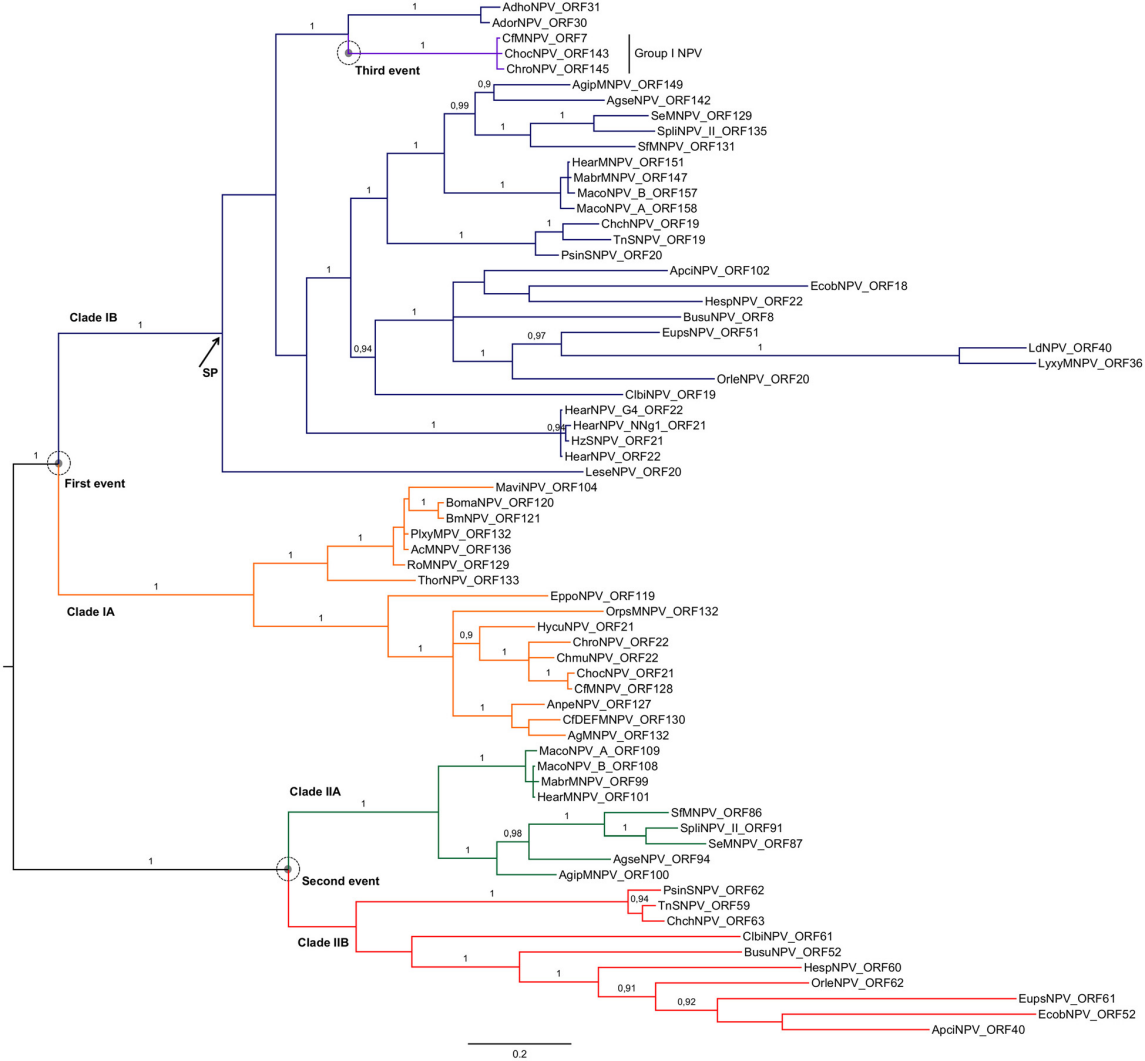
**Bayesian Phylogram based on P26 deduced amino acid sequences.** The clustering pattern suggests the occurrence of three independent *p26* gene acquisition events that are marked by circles. The arrow indicates the node where occurred the signal peptide acquisition. Numbers at branches indicate the Bayesian posterior probabilities (value > 0.9). The tree was rooted by midpoint approach. The scale bar indicates the number of substitutions per site.

The phylogenetic tree obtained by Bayesian Phylogenetic Inference (BPI) using the *p26* copies found in all Alphabaculovirus genomes so far sequenced showed four clearly defined clades (IA, IB, IIA and IIB) nested within the larger clades I and II (Figure 3). The clade IA contains *p26* copies from Group I Alphabaculoviruses and clade II from Group II Alphabaculoviruses. Clade IB contains *p26* copies also from Group II Alphabaculoviruses, except for a monophyletic group with CfMNPV_ORF31, ChocMNPV_ORF143 and ChroNPV_ORF145 from Group I Alphabaculoviruses. Clades I (IA and IB) and II correlate with the position of the genes in the genome, where clade I contains the *p26* copies adjacent to the *p10* gene (position 1), except for CfMNPV_ORF31, ChocMNPV_ORF143 and ChroNPV_ORF145, which are adjacent to *ptp1* and *ptp2* (position 3), and clade II contains the *p26* copies that are adjacent to *iap-2* (position 2). This clustering pattern suggests the occurrence of three independent acquisition events of the *p26* gene by baculoviruses. The first acquisition event occurred in position 1 of the common ancestral genome of all baculoviruses containing this gene. The second acquisition event generated the *p26* second copy in position 2 of the Group II Alphabaculovirus genome with two *p26* homologs. Finally, the third acquisition event occurred in CfMNPV, ChocNPV and ChroNPV (Group I Alphabaculoviruses), inserting the second *p26* copy in position 3.

The acquisition of baculovirus genes may be the results of duplication and horizontal gene transfer by transposable element and/or homologous recombination. The second acquisition event probably occurred by horizontal gene transfer. In this case, the gene duplication hypothesis can be refuted, since the similarity between *p26* copies in the same virus is low (less than 30% identity). Furthermore, clades I and II are clearly separated, indicating that the *p26* copies did not originate from a common, recent ancestor. In the third acquisition event, there is high similarity between the second *p26* copy of the Group I Alphabaculoviruses and first *p26* copy of the Group II Alphabaculoviruses, grouping these genes in the same clade (clade IB). Therefore, the second *p26* copy of the Group I Alphabaculoviruses may have been acquired from a Group II Alphabaculovirus.

The isoelectric point (pI) and molecular weight (MW) of the deduced P26 protein from Alphabaculoviruses with two *p26* copies were calculated, and are presented in the context of their genomic positioning (Table 2). P26 protein from position 1 showed an average pI of 6.7 ± 0.76 and average MW of 30,526 ± 2,387; position 2 P26 proteins have an average pI of 8.2 ± 0.71 and average MW of 27,354 ± 1,306; and position 3 P26 proteins have an average pI of 9.5 ± 0.07 and an average MW of 30,113 ± 24. The mean scores were examined using the Student’s t-test and the pI average showed a significant difference (p < 0.05) between the three positions. The isoelectric point difference suggests that the P26 sequence in position 3 exhibits more basic amino acids than in those in positions 1 and 2.

The presence and location of signal peptide cleavage sites in P26A and P26B amino acid sequences from PsinSNPV were analysed using SignalP v.4.1. The P26A protein showed a signal peptide cleavage site at amino acid residues 1 to 21 and the cleavage site (IMS-T) between amino acids 21 and 22 (Additional file 6). However, the signal peptide cleavage site was absent in the P26B protein. Signal peptides direct the proteins to their proper cellular and extracellular locations. The export of proteins occurs via the secretory pathway, where proteins labeled by an N-terminal signal sequence are translocated across the cytoplasmic membrane, whereafter the N-terminal signal peptide is usually cleaved by an extracellular signal peptidase.

The signal peptide cleavage sites were predicted for other P26 proteins of Alphabaculoviruses with complete sequenced genomes and the results are shown in Table 2. The presence or absence of signal peptides in P26 proteins correlates with the clustering obtained in the phylogenetic analysis, where only P26 proteins from clade IB possess signal peptides. However, four sequences belonging to this clade, LeseNPV_ORF20, EcobNPV_ORF18, OrleNPV_ORF20 and ChchNPV_ORF19, do not possess a signal peptide. The presence or absence of the signal peptide may have led to the differential results found in predicted molecular weight and isoelectric points between the P26 proteins analyzed. The presence of a signal peptide in the first *p26* copy of Group II Alphabaculoviruses and in the second *p26* copy of Group I Alphabaculoviruses suggests that this domain was acquired from a common ancestor of these viruses. Although the function of P26 is not well understood, the signal peptide may lead to differences in activity of the clade IB proteins compared to the P26 from other clades, which warrants further investigation.

## Conclusions

In summary, the complete PsinSNPV-IE genome sequence is apparently different from other baculoviruses sequenced so far. The genome does not contain the typical baculovirus *hrs* and contains two ORFs with predicted transmembrane domains that are unique to PsinSNPV. The PsinSNPV genome, however, exhibits high sequence similarities and co-linearity to the closely related ChchNPV and TnSNPV. The PsinSNPV genome contains two *p26* copies and a phylogenetic analysis of P26 sequences of Alphabaculoviruses showed three potential acquisition events of these genes within the *Baculoviridae* family. One of the clades comprises P26 protein with a signal peptide, indicating a possible distinct function from other classes of P26 protein. However, further investigations are needed for a better understanding of this protein in baculoviruses. This research reports the first completely sequenced genome of a strain of PsinSNPV, a currently little known baculovirus. It is anticipated that this data will both promote advances in investigations of its molecular biology and gene function and accelerate its development as a biocontrol agent.

## Methods

### Virus and viral DNA extraction

The *Pseudoplusia includens* SNPV – IE isolate, donated by Dr. Flávio Moscardi, Embrapa Soja (Londrina-PR), was obtained from an infected *C. includens* larva collected on soybean from a farm in Iguaraçu - PR-Brazil in 2007, and has been deposited in the Invertebrate Virus Collection at Embrapa Genetic Resources and Biotechnology. This isolate is listed in the Brazilian AleloMicro Information System under accession code BRM 005106. Viral OBs from *C. includens* larval cadavers were purified by differential centrifugation according to procedures described by Maruniak [59]. DNA was extracted from ODVs as described previously [24,25]. The quality of the extracted DNA was determined by 0.5% agarose gel electrophoresis and quantified using a Qubit v. 2.0 Fluorometer (Invitrogen) according to the manufacturer’s instructions.

### DNA sequence determination

The genome DNAs of seven PsinSNPV isolates (IA to IG), which have been investigated in our laboratory [24,25], were sequenced using the shotgun approach and were performed using the 454 Roche GS FLX – Titanium instrument at the Federal District (DF, Brazil) High-Performance Genome Center. Raw reads were processed using Newbler v. 2.8 (Roche Applied Science) and Biopieces scripts were used to create the fastq files. FastQC was used for quality assessment and Coral v. 1.4 [60] was used to correct sequencing errors. PrinSeq v. 0.20.3 [61] (Preprocessing and Information of Sequences) was applied to trim low quality reads (Phred ≤ 20) and to remove short sequences (length ≤ 50 bp). An error probability of 0.1% was allowed and 0.27% of the overall reads allowed to contain the ambiguous base ‘N’. The Phred score was measured and the mean sequence quality >30 was estimated, exhibiting an accuracy of 99.9% (Additional file 1). *De novo* assembly of reads from all isolates were carried out together using the MIRA assembler v. 3.4.1.1 [62] resulting in a single contig of 148,729 bp. This scaffold was used to map the trimmed reads from the isolate PsinSNPV-IE, resulting in a final assembly for this single isolate, with a minimum coverage of 30X, representing its complete genomic sequence. SNPS and indels present in the assembled sequence were observed, since the sequenced isolate was not plaque purified, representing natural genotypic variation within PsinSNPV-IE [25]. The final sequence was the 50% majority rule consensus of the PsinSNPV-IE reads. The polyhedrin gene was identified and the PsinSNPV circular nucleotide sequence was determined. An *in silico Bam*HI, *Eco*RI, *Hin*dIII and *Pst*I endonuclease restriction map was constructed using Geneious R6 v. 6.0.5 (Biomatters, Auckland, New Zealand) and was compared with DNA restriction profiles of PsinSNPV-IE determined previously [25].

### Sequence data bioinformatics analysis

ORF prediction was carried out with ORF Finder (National Center for Biotechnology Information -NCBI) and Geneious (v. 6.0.5.). ATG-initiated ORFs encoding more than 50 amino acids with minimal overlaps were selected. Relevant ORFs were aligned against ChchNPV and TnSNPV genomes, and PsinSNPV-IE gene models confirmed using Artemis software [63]. The BLASTx algorithm [64] was used to annotate the predicted ORFs. Percentage identities between homologous genes were obtained by alignment of the proteins from whole genomes using the tBLASTn program [64]. Global alignment of the PsinSNPV genome against other baculovirus genomes was performed and the syntenic map constructed using Mauve alignment v. 2.0 implemented in the Geneious v. 6.0.3 package [65,66]. A dot-plot analysis was applied to compare the PsinSNPV genome against ChchNPV, TnSNPV, *Mamestra configurata* (Maco) NPV-B and *Autographa californica* (Ac) MNPV using LBDotView v. 1.0 software [67].

Deduced protein sequences were also analysed using SignalP 4.1 Server [68] (http://www.cbs.dtu.dk/services/SignalP/) and TMHMM (TransMembrane prediction using Hidden Markov Models) Server v. 2.0 [69,70] for prediction of signal peptide cleavage sites and Transmembrane (TM) helices, respectively.

### P26 phylogenetic analysis

PsinSNPV P26a and P26b amino acid sequences were aligned using MUSCLE v. 3.5 software [71] against the corresponding amino acid sequences from other baculoviruses with sequenced genome (Table 2). A statistical model-fitting approach was conducted using ProtTest [72] and the LG model [73] was selected as best-fit model for the P26 alignment. Bayesian phylogenetic inference (BPI) was conducted using MrBayes v. 3.0b4 [74]. Because MrBayes does not support the LG model of evolution, likelihood settings were set to aamodel = mixed rates = invgamma, which allowed the best model of substitution to be selected as a parameter of the analysis [75]. Five Markov chains were run for 600,000 generations (p < 0.01), sampling every 100 generations. The first 25% of the trees obtained in the analysis were discarded as burn-in before computing the consensus tree.

### Availability of supporting data

[GenBank: KJ631622].

## Additional files

Additional file 1:
**Results of the read-quality check.** (A) Basic statistics of the raw and trimmed data. (B) Phred quality score distribution in trimmed reads. Additional file 2:
**PsinSNPV genome features and comparison of 141 putative ORFs with homologs in Alphabaculoviruses.** Gray lines indicate the core genes. Additional file 3:
**Multiple genome alignment of PsinSNPV with other Alphabaculoviruses.** (A) PsinSNPV genome was aligned with (B) ChchNPV, (C) TnSNPV, (D) MacoNPV-B and (E) AcMNPV using Mauve software. Local collinear blocks (LCB) are shown by boxes with identical colors and represent the homologous regions shared by two or more genomes. LCBs below the horizontal black line represent the reverse complement of the PsinSNPV LCB. Additional file 4:
**Hydrophobicity plot of the photolyase amino acid sequence of PsinSNPV.** Alignment of PsinSNPV-IE, TnSNPV, ChchNPV - PHR1, −PHR2 and *Drosophila melanogaster* photolyase protein sequences was performed using MUSCLE v. 3.5 software. The most hydrophobic residues are colored in red and the most hydrophilic in blue. Conserved tryptophans (W) are indicated by a blue box and non-conserved by a red box. Additional file 5:
**Predicted transmembrane helices from the deduced amino acid sequences of the ORFs unique to PsinSNPV.** The predicted transmembrane helices from predicted protein sequence of the PsinSNPV (A) ORF-5 and (B) ORF-8 using TMHMM Server v. 2.0 program are shown in red in Psin5 between the 79 to101 and 148 to 170 amino acids and in Psin8 within the 69 to 91 amino acids. Additional file 6:
**P26 signal peptide predicted using SignalP v. 4.1.** The green line indicates the motif with high probability of comprising the signal peptide (position 1–21, mean S-score = 0.791). The cleavage site was predicted to be between Ser21 and Thy22 (IMS-TQ ; D score = 0.629).
