# Supplementary material for: The genome sequence of Pseudoplusia includens single nucleopolyhedrovirus and an analysis of p26 gene evolution in the baculoviruses
Source: BMC Genomics. 2015 Feb 25;16(1):127. doi: 10.1186/s12864-015-1323-9 (PMC4346127; doi:10.1186/s12864-015-1323-9)

(A)

Basic Statistics – raw data
Sequences: 38,281
Total bases: 20,751,970
Mean sequence length: 542.10 ± 67.48 bp
Minimum length: 55 bp
Maximum length: 1,200 bp
Mean GC content: 40.56 ± 4.07 %
Sequences with N: 15,645 (40.87 %)
Max percentage of Ns per sequence: 36 %

Basic Statistics – trimmed data
Sequences: 33,596
Total bases: 11,787,493
Mean sequence length: 350.86 ± 121.32 bp
Minimum length: 50 bp
Maximum length: 500 bp
Mean GC content: 39.74 ± 5.15 %
Sequences with N: 91 (0.27 %)
Max percentage of Ns per sequence: 1%

(B)

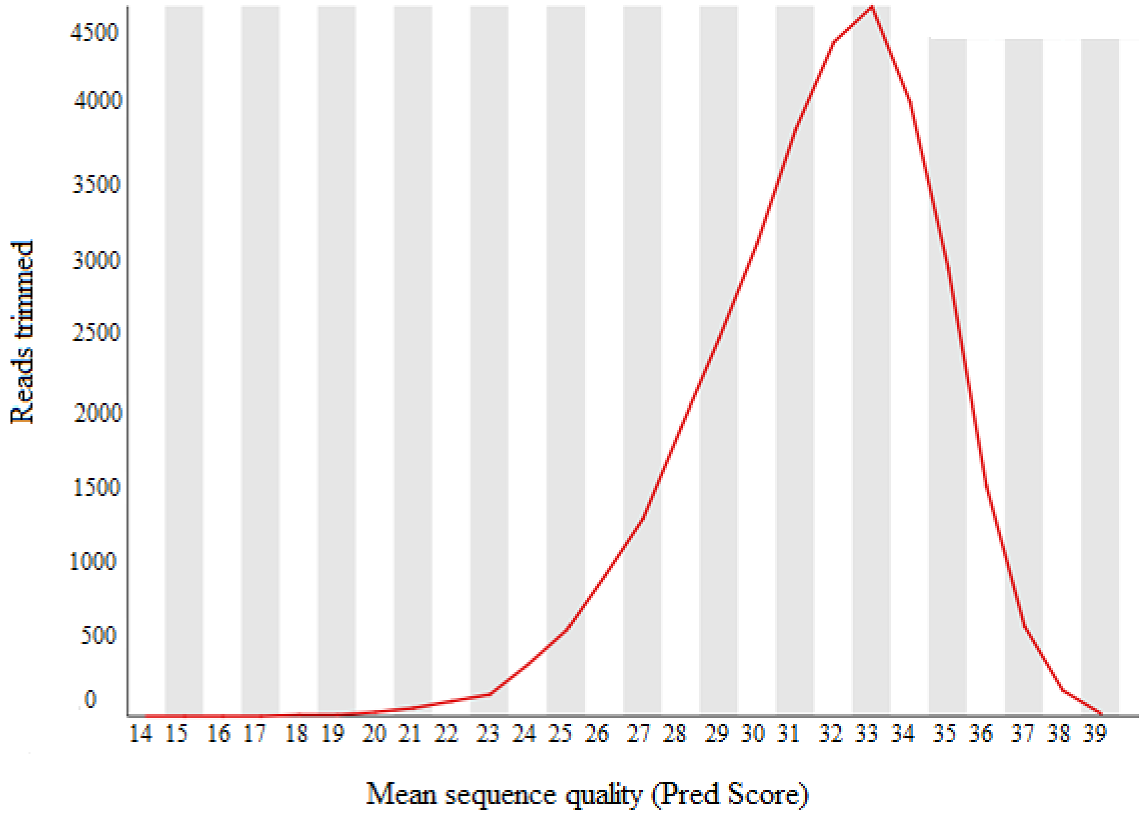

Supplement: Additional file 1: — Results of the read-quality check. (A) Basic statistics of the raw and trimmed data. (B) Phred quality score distribution in trimmed reads. [file 12864_2015_1323_MOESM1_ESM.pdf]
