# Supplementary material for: The genome sequence of Pseudoplusia includens single nucleopolyhedrovirus and an analysis of p26 gene evolution in the baculoviruses
Source: BMC Genomics. 2015 Feb 25;16(1):127. doi: 10.1186/s12864-015-1323-9 (PMC4346127; doi:10.1186/s12864-015-1323-9)
Supplement: Additional file 2: — PsinSNPV genome features and comparison of 141 putative ORFs with homologs in Alphabaculoviruses. Gray lines indicate the core genes. [file 12864_2015_1323_MOESM2_ESM.pdf]

PsinSNPV genome features and comparison of 141 putative ORFs with homologs in Alphabaculoviruses

PsinSNPV				Homologs (ORF; %ID)							
ORF	Name	Position	Length (nt)	AcMNPV		ChchNPV		TnSNPV		MacoNPV-B	
1	*polh*	1→741	741	8	88.84	1	96.34	1	95.12	1	92.28
2	*orf1629*	738←1,937	1,200	9	39.6	2	76.98	2	62.23	2	28.16
3	*pK1*	1,961→2,779	819	11	42.57	3	95.22	3	92.65	3	50.00
4	*hoar*	2,804←4,822	2,019	-		4	57.20	4	55.17	4	22.62
5	*unknown*	5,401→5,916	516	-		-		-		-	
6	*unknown*	6,016←6,195	180	152	59.62	-		-		-	
7	*unknown*	6,338→7,312	975	-		-		7	31	-	
8	*unknown*	7,325→7,624	300	-		-		-		-	
9	*odv-e56/pif-5*	7,721→8,797	1,077	148	51.22	6	75.98	8	81.84	6	51.57
10	*me53*	8,896←9,999	1,104	139	24.57	7	89.67	9	86.68	7	40.72
11	*exon-0/ie-0*	10,298→11,248	951	141	34.72	8	79.25	10	74.05	167	39.08
12	*p49*	11,265→12,689	1,425	142	51.44	11	96.84	11	96.42	166	62

13	*odv-e18*	12,699→12,944	246	143	56.9	12	53.09	12	54.32	165	56.7
14	*odv-ec27*	12,985→13,866	882	144	52.54	13	93.81	13	89.19	164	61.72
15	*unknown*	13,872→14,153	282	145	46.75	14	89.25	14	82.80	163	55
16	*unknown*	14,203←14,826	624	146	33.33	15	80.19	15	76.33	162	44
17	*ie-1*	14,865→17,207	2,343	141a	34.72	16	66.38	16	82.60	161	36.10
18	*p74/pif-0*	17,320→19,296	1,977	138	53.54	17	91.34	17	90.47	159	54.68
19	*p10*	19,350←19,616	267	-		18	86.67	18	90.91	158	51.35
20	*p26a*	19,668←20,519	852	136	33.73	19	79.93	19	78.09	157	57.32
21	*unknown*	20,680→20,979	300	29	37	20	92.93	20	95.96	156	53
22	*lef-6*	21,007←21,478	472	28	35	21	69.51	21	75.30	155	44.03
23	*dbp*	21,49←22,473	984	25	30.26	22	74.14	22	84.29	154	32.64
24	*unknown*	22,572→23,003	432	26	33	23	61.70	23	64.79	153	37
25	*unknown*	23,058→24,329	1,272	-		24	48.36	-		-	
26	*unknown*	24,423←25,165	743	34	32.39	25	67.33	24	62.19	152	59
27	*vubi*	25,175→25,408	234	35	68.42	26	97.40	25	100.00	151	70.15

28	*unknown*	25,405→25,635	231	-		27	80.82	25b	74.32	150	34
29	*39K*	25,719←26,696	978	36	34.04	28	73.93	26	71.56	149	45.21
30	*lef-11*	26,671←27,138	468	37	41.38	29	77.42	27	76.36	148	60.00
31	*unknown*	27,090←27,821	732	38	60.64	30	83.51	28	85.02	147	71
32	*unknown*	27,962←28,489	528	63	22.75	31	58.29	29	68.00	-	
33	*bro-a*	28,758→30,152	1,395	-		55	51	-		20	53.54
34	*p47*	30,905←32,098	1,194	40	54.76	33	92.73	31	92.70	144	65.00
35	*unknown*	32,298→33,053	756	-		34	53.11	-		-	
36	*unknown*	33,168←33,758	591	-		36	37.61	-		-	
37	*lef-8*	34,016←36,844	2,829	50	53.10	37	80.91	33	80.91	140	67.15
38	*bjdp*	36,868→37,905	1,038	-		38	76.19	34	80.71	139	30.11
39	*iap-3*	37,972←38,820	849	27	31.60	39	71.48	35	74.37	138	33.33
40	*unknown*	38,978→39,154	177	-		-		36	52.54	-	
41	*unknown*	39,151←39,759	609	-		40	68.98	37	64.22	137	32
42	*unknown*	39,821→40,237	417	53	48.48	41	94.20	38	91.39	136	59

43	*unknown*	40,242←41,354	1,113	-		42	64.81	39	63.93	135	38
44	*unknown*	41,395←41,628	234	-		43	81.82	40	81.82	134	47
45	*lef-10*	41,588→41,815	228	53a	48.48	44	85.53	41	94.67	133	62.67
46	*vp1054*	41,676→42,680	1,005	54	36.96	45	86.90	42	83.93	132	57.19
47	*unknown*	42,804→43,040	237	-		46	67.06	43	67.03	131	49
48	*unknown*	42,916→43,314	399	-		47	73.64	44	80.30	130	50
49	*unknown*	43,556→44,068	513	57	44.03	48	82.35	45	82.46	129	45
50	*unknown*	44,076←44,574	499	59	50.91	49	76.27	46	69.05	128	76
51	*unknown*	44,595←44,871	277	60	50.82	50	94.03	47	95.52	127	60
52	*fp25k*	45,140←45,828	689	61	62.50	51	86.85	-		116	76.88
53	*lef-9*	45,910→47,397	1,488	62	68.72	52	96.51	49	96.51	123	77.41
54	*unknown*	47,553←47,762	210	-		53	53	-		-	
55	*unknown*	48,574→48,828	255	-		56	80.00	52	76.47	-	
56	*unknown*	48,835→49,224	390	-		57	93.02	53	93.02	115	62
57	*dnapol*	49,246←52,425	3,18	65	42.95	58	80.91	54	81.72	114	60.86

58	*desmoplakin*	52,424→54,556	2,133	66	39.58	59	73.64	55	77.64	113	65.31
59	*lef-3*	54,672←56,075	1,404	67	25.83	60	74.93	56	72.51	112	32.52
60	*unknown*	56,074→56,469	396	68	45.83	61	75.57	57	88.55	111	62
61	*iap-2*	56,521→57,414	894	71	25.71	62	79.22	58	72.85	109	45.21
62	*p26b*	57,460→58,197	738	-		63	83.68	59	85.36	108	32.92
63	*vcath*	58,313←59,350	1,038	127	56.35	64	93.93	60	92.49	28	62.87
64	*chitinase*	59,464→61,173	1,71	126	68.23	65	85.59	61	83.66	19	67.90
65	*unknown*	61,291→62,037	747	-		-		62	61.75	-	
66	*pcna*	62,009←62,797	789	49	24.09	66	70.61	63	73.28	-	
67	*gp37*	62,929→63,747	819	64	60.50	67	93.01	64	91.91	32	69.77
68	*phr*	63,844→65,376	1,533	-		68	60.28	65	59.00	-	
69	*bro-b*	65,452←66,912	1,461	-		69	71	108	40.62	58	41.52
70	*unknown*	67,059←67,344	286	-		71	51.02	66	51.92	-	
71	*he65*	68,173→68,877	705	105	40.12	73	82.21	-		27	37.94
72	*ctl*	68,972→69,121	150	3	45	74	85.71	-		106	66

73	*unknown*	69,153←69,539	387	84	37.50	75	39.42	-		-	
74	*vlf-1*	69,648←70,823	1,176	77	71.90	76	81.95	70	81.95	105	70.33
75	*unknown*	70,820←71,194	375	78	33	77	78.23	71	78.57	104	41
76	*gp41*	71,217←72,176	960	80	54.40	78	99.67	72	95.33	103	61.87
77	*unknown*	72,118←72,834	717	81	50.70	79	83.25	74	82.68	102	66
78	*tlp20*	72,722←73,441	720	-		80	71.37	75	57.19	101	68
79	*vp91/p95*	73,410→75,891	2,482	83	39.71	81	77.18	76	78.41	100	49
80	*vp39*	75,970←76,980	1,011	89	40.80	82	93.13	77	93.73	98	50.16
81	*lef-4*	76,955→78,370	1,416	90	41.37	83	78.97	78	76.06	97	55.70
82	*p33*	78,466←79,227	762	92	51.16	84	92.03	79	93.23	95	66
83	*p18*	79,220→79,702	483	93	52.23	85	97.50	80	98.12	94	79
84	*odv-e25*	79,699→80,358	660	94	39.25	86	72.89	81	73.64	93	66.36
85	*helicase*	80,480←84,109	3,63	95	40.32	87	84.60	82	81.59	92	59
86	*odv-e28/pif-4*	84,036→84,584	549	96	52.45	88	91.67	83	81.40	91	67
87	*unknown*	84,618→85,226	609	-		89	58.82	84	57.06	-	

88	*unknown*	85,253→85,483	231	-		90	51.25	-		-	
89	*38K*	85,514←86,470	957	98	40.84	91	84.54	86	86.98	87	63
90	*lef-5*	86,363→87,226	864	99	41.24	92	72.47	87	72.07	86	53
91	*p6.9*	87,220←87,492	273	100	40.9	93	83	88	74.6	85	54.2
92	*p40*	87,579←88,721	1,143	101	42.52	94	83.51	89	87.93	84	63
93	*p12*	88,744←88,995	252	102	31.33	95	86.00	90	90.11	83	56
94	*p48/p45*	89,098←90,237	1,14	103	46.46	96	91.56	91	89.97	82	69
95	*p87/vp80*	90,272→91,981	1,71	-		97	74.87	92	82.67	81	45.54
96	*odv-ec43*	92,145→93,221	1,077	109	47.57	99	99.44	94	99.15	79	60
97	*unknown*	93,257→93,535	279	-		100	55.68	95	84.78	78	57
98	*odv-e66*	93,579←95,606	2,028	46	36.08	101	79.91	96	80.09	77	57.94
99	*p13*	95,692←96,606	915	-		102	89.47	97	85.53	75	55.52
100	*unknown*	96,925→97,374	450	-		103	57.43	99	40.26	-	
101	*unknown*	97,403←98,398	996	-		104	40.06	100	44.41	-	
102	*unknown*	98,569←98,961	393	-		105	53.85	-		-	

103	*unknown*	99,155→100,27	1,116	-		106	63.95	101	67.44	71	35
104	*unknown*	100,279←100,965	687	106	62.50	107	88.21	102	83.77	70	65
105	*unknown*	101,008←102,591	1,584	-		108	73.91	103	77.14	69	25
106	*unknown*	102,683←103,636	954	-		109	53.63	104	48.73	-	
107	*pif-3*	103,738←104,376	639	115	44.25	110	89.29	105	86.15	67	44
108	*unknown*	104,461←104,889	429	-		111	49.64	-		66	24
109	*unknown*	105,014→105,589	576	-		112	87.30	107	86.24	118	34
110	*sod29*	105,654→106,109	456	33	74.50	115	93.33	109	91.33	65	80.67
111	*unknown*	106,182←106,823	642	-		116	43.37	110	44.19	-	
112	*unknown*	106,979←107,251	273	-		117	70.30	111	67.37	-	
113	*unknown*	107,485←107,997	513	-		118	61.76	112	64.71	-	
114	*dUTPase*	108,162←108,626	465	-		119	43.51	-		73	36.61
115	*calyx/pep*	108,903→109,892	990	131	43	121	98.06	113	43.40	61	68
116	*rr2*	109,984←110,937	954	-		122	92.45	114	87.42	52	58.73
117	*unknown*	111,057→111,455	399	-		123	88.64	115	87.12	51	36

118	*unknown*	111,483→112,607	1,125	-		124	88.50	116	89.63	-	
119	*unknown*	112,649←113,938	1,29	18	23.16	125	75.34	117	78.21	50	42
120	*unknown*	113,940→114,320	381	-		126	69.92	118	69.84	49	40
121	*alkexo*	114,292→115,536	1,245	133	37.50	127	81.55	119	77.48	48	42
122	*unknown*	115,555←116,295	741	-		128	78.97	120	72.18	47	37
123	*fgf*	116,574→117,686	1,113	35	31.67	130	62.31	122	68.94	46	39.52
124	*pif-1*	117,791←119,347	1,557	119	49.05	131	89.42	123	88.40	44	50
125	*unknown*	119,338←119,970	633	-		132	63.58	124	63.10	-	
126	*gp16*	119,992←120,279	288	130	33.68	133	73.68	125	88.42	10	44.09
127	*p24*	120,215←121,030	816	129	48.15	134	80.40	126	84.90	11	57.78
128	*unknown*	121,156→121,560	405	-		135	68.57	127	62.59	-	
129	*lef-2*	121,490→122,161	672	6	37.62	136	75.66	128	80.00	13	47.66
130	*38.7 K*	122,255←123,394	1,14	13	45.45	137	70.00	129	74.26	31	37
131	*lef-1*	123,440←124,081	642	14	44.39	138	79.91	130	88.32	30	51.15
132	*unknown*	124,121→124,582	462	-		139	60.00	131	52.98	-	

133	*ptp2*	124,584←125,081	498	-		140	85.28	132	87.12	33	48.65
134	*egt*	125,241→126,806	1,566	17	49.31	141	93.78	133	90.79	34	65.83
135	*unknown*	127,010→127,588	579	-		142	86.26	135	79.12	35	33
136	*unknown*	127,771←130,542	2,772	-		143	75.19	-		37	33
137	*pkip*	131,117→131,620	504	-		146	85.63	139	78.44	40	39.52
138	*arif-1*	131,695←132,675	981	21	26.06	147	59.27	140	58.91	42	34.15
139	*pif-2*	132,670→134,091	1,422	22	59.33	148	93.15	141	88.77	43	63
140	*f protein*	134,217←136,193	1,977	23	21.90	150	94.10	143	92.44	8	41
141	*rr1*	136,539←138,884	2,346	-		151	83.80	144	83.92	168	54.12

Gray lines indicate the core genes.
