# Supplementary figures and images for: The genome sequence of Pseudoplusia includens single nucleopolyhedrovirus and an analysis of p26 gene evolution in the baculoviruses
Source: BMC Genomics. 2015 Feb 25;16(1):127. doi: 10.1186/s12864-015-1323-9 (PMC4346127; doi:10.1186/s12864-015-1323-9)

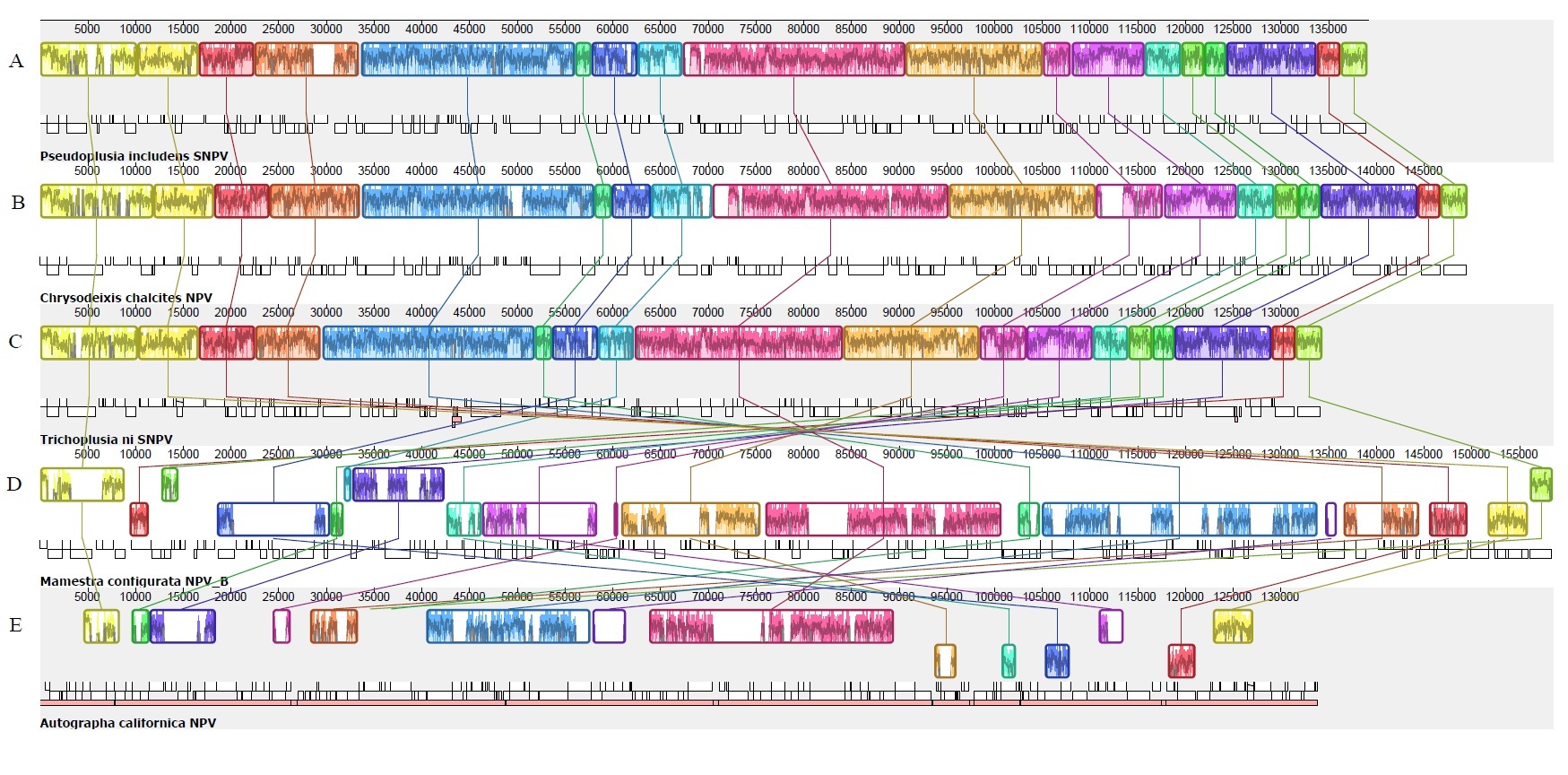

Supplement: Additional file 3: — Multiple genome alignment of PsinSNPV with other Alphabaculoviruses. (A) PsinSNPV genome was aligned with (B) ChchNPV, (C) TnSNPV, (D) MacoNPV-B and (E) AcMNPV using Mauve software. Local collinear blocks (LCB) are shown by boxes with identical colors and represent the homologous regions shared by two or more genomes. LCBs below the horizontal black line represent the reverse complement of the PsinSNPV LCB. [file 12864_2015_1323_MOESM3_ESM.jpeg]

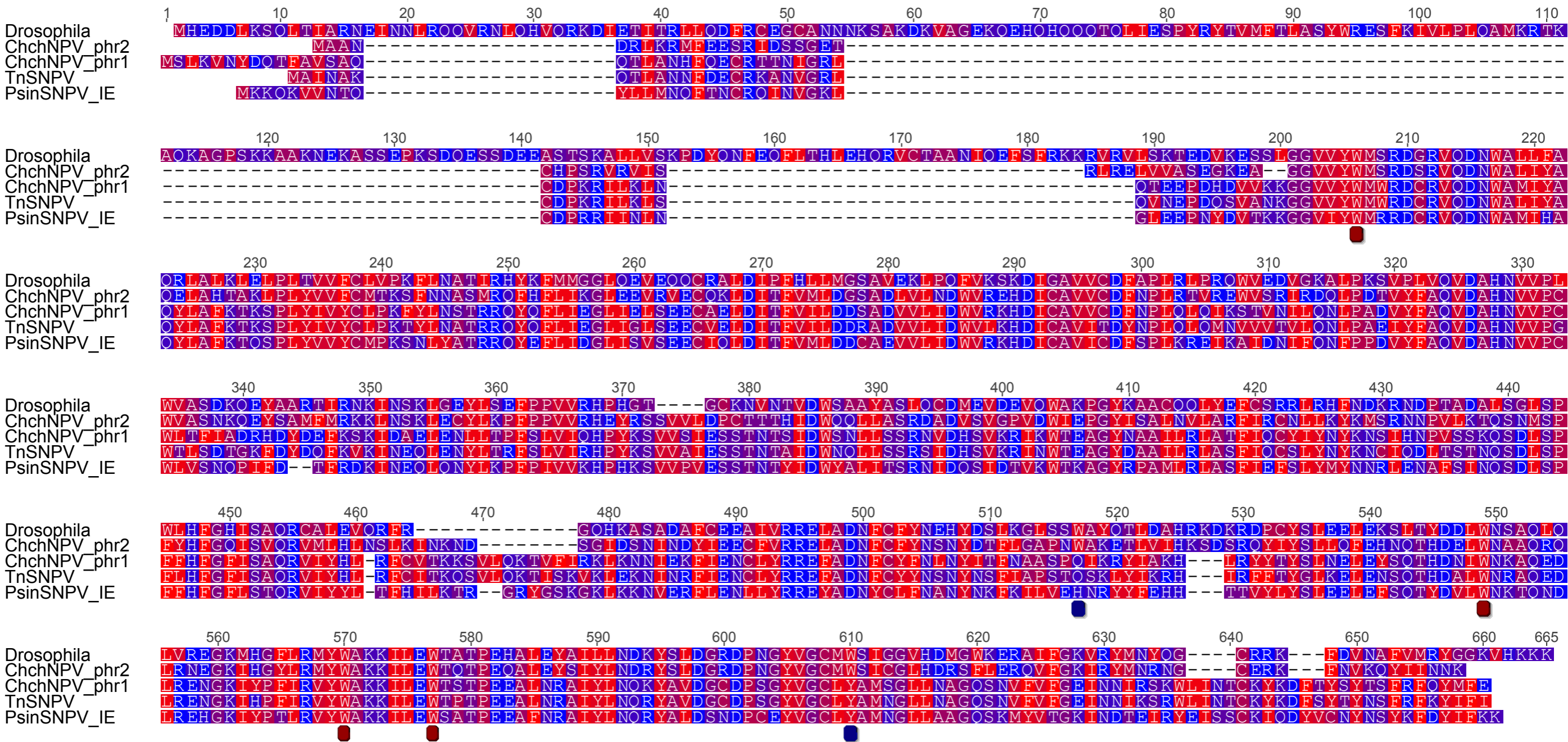

Supplement: Additional file 4: — Hydrophobicity plot of the photolyase amino acid sequence of PsinSNPV. Alignment of PsinSNPV-IE, TnSNPV, ChchNPV - PHR1, −PHR2 and Drosophila melanogaster photolyase protein sequences was performed using MUSCLE v. 3.5 software. The most hydrophobic residues are colored in red and the most hydrophilic in blue. Conserved tryptophans (W) are indicated by a blue box and non-conserved by a red box. [file 12864_2015_1323_MOESM4_ESM.pdf]

**A) Psin5**

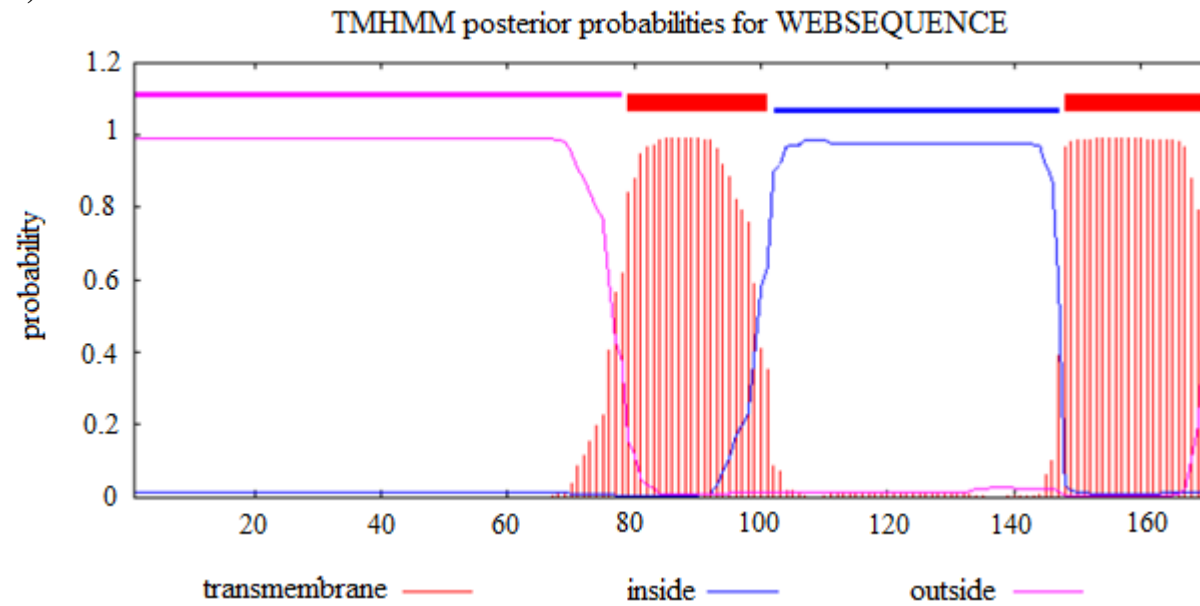

**B) Psin8**

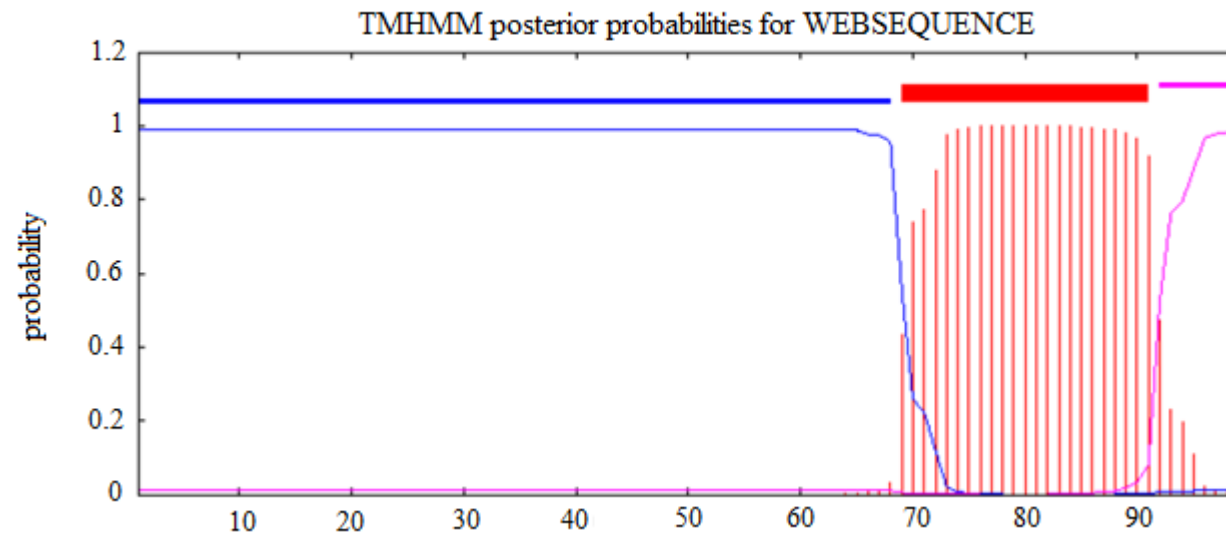

Supplement: Additional file 5: — Predicted transmembrane helices from the deduced amino acid sequences of the ORFs unique to PsinSNPV. The predicted transmembrane helices from predicted protein sequence of the PsinSNPV (A) ORF-5 and (B) ORF-8 using TMHMM Server v. 2.0 program are shown in red in Psin5 between the 79 to101 and 148 to 170 amino acids and in Psin8 within the 69 to 91 amino acids. [file 12864_2015_1323_MOESM5_ESM.pdf]

SignalP-4.1 prediction (euk networks): Sequence

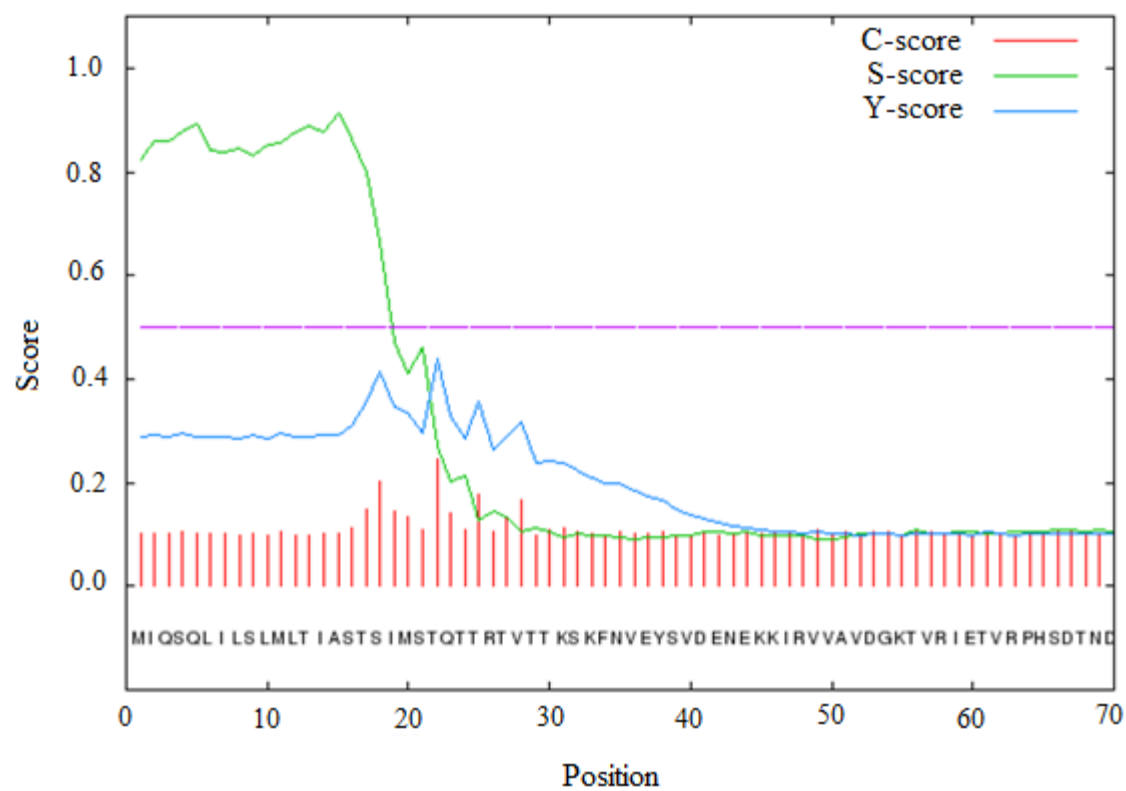

Supplement: Additional file 6: — P26 signal peptide predicted using SignalP v. 4.1. The green line indicates the motif with high probability of comprising the signal peptide (position 1–21, mean S-score = 0.791). The cleavage site was predicted to be between Ser21 and Thy22 (IMS-TQ ; D score = 0.629). [file 12864_2015_1323_MOESM6_ESM.pdf]
